# Maternal Pyrroloquinoline Quinone Supplementation Improves Offspring Liver Bioactive Lipid Profiles throughout the Lifespan and Protects against the Development of Adult NAFLD

**DOI:** 10.3390/ijms23116043

**Published:** 2022-05-27

**Authors:** Ashok Mandala, Evgenia Dobrinskikh, Rachel C. Janssen, Oliver Fiehn, Angelo D’Alessandro, Jacob E. Friedman, Karen R. Jonscher

**Affiliations:** 1 Harold Hamm Diabetes Center, University of Oklahoma Health Sciences Center, Oklahoma City, OK 73104, USA; ashok-mandala@ouhsc.edu (A.M.); rachel-janssen@ouhsc.edu (R.C.J.); jed-friedman@ouhsc.edu (J.E.F.); 2 Section of Neonatology, Department of Pediatrics, University of Colorado Anschutz Medical Campus, Aurora, CO 80045, USA; evgenia.dobrinskikh@cuanschutz.edu; 3 Genome Center-Metabolomics, University of California Davis, Davis, CA 95616, USA; ofiehn@ucdavis.edu; 4 Department of Medicine, University of Colorado Anschutz Medical Campus, Aurora, CO 80045, USA; angelo.dalessandro@cuanschutz.edu; 5 Department of Physiology, University of Oklahoma Health Sciences Center, Oklahoma City, OK 73104, USA; 6 Department of Biochemistry and Molecular Biology, University of Oklahoma Health Sciences Center, Oklahoma City, OK 73104, USA

**Keywords:** pyrroloquinoline quinone, pediatric NAFLD, untargeted lipidomics, very long-chain ceramides, hepatic PC/PE ratio, fatty acid oxidation

## Abstract

Maternal obesity and consumption of a high-fat diet significantly elevate risk for pediatric nonalcoholic fatty liver disease (NAFLD), affecting 10% of children in the US. Almost half of these children are diagnosed with nonalcoholic steatohepatitis (NASH), a leading etiology for liver transplant. Animal models show that signs of liver injury and perturbed lipid metabolism associated with NAFLD begin in utero; however, safe dietary therapeutics to blunt developmental programming of NAFLD are unavailable. Using a mouse model of maternal Western-style diet (WD), we previously showed that pyrroloquinoline quinone (PQQ), a potent dietary antioxidant, protected offspring of WD-fed dams from development of NAFLD and NASH. Here, we used untargeted mass spectrometry-based lipidomics to delineate lipotoxic effects of WD on offspring liver and identify lipid targets of PQQ. PQQ exposure during pregnancy altered hepatic lipid profiles of WD-exposed offspring, upregulating peroxisome proliferator-activated receptor (PPAR) α signaling and mitochondrial fatty acid oxidation to markedly attenuate triglyceride accumulation beginning in utero. Surprisingly, the abundance of very long-chain ceramides, important in promoting gut barrier and hepatic function, was significantly elevated in PQQ-treated offspring. PQQ exposure reduced the hepatic phosphatidylcholine/phosphatidylethanolamine (PC/PE) ratio in WD-fed offspring and improved glucose tolerance. Notably, levels of protective n − 3 polyunsaturated fatty acids (PUFAs) were elevated in offspring exposed to PQQ, beginning in utero, and the increase in n − 3 PUFAs persisted into adulthood. Our findings suggest that PQQ supplementation during gestation and lactation augments pathways involved in the biosynthesis of long-chain fatty acids and plays a unique role in modifying specific bioactive lipid species critical for protection against NAFLD risk in later life.

## 1. Introduction

Pediatric nonalcoholic fatty liver disease (NAFLD) is on the rise and is the leading cause of chronic liver disease in overweight and obese children, adolescents, and young adults worldwide [1,2]. NAFLD represents a spectrum of liver disorders ranging from simple steatosis to inflammatory nonalcoholic steatohepatitis (NASH) with or without fibrosis [3]. The pathogenesis of pediatric NAFLD is multifactorial and may be distinct from the adult form of the disease due to its rapid onset and histopathological changes within the liver [4,5,6]. Pediatric NAFLD can initiate prior to obesity [6,7] and leads to hepatic as well as systemic insulin resistance [5].

Compelling evidence demonstrates that the perinatal environment has direct effects on offspring vulnerability to disease later in life. Fetal exposure to a suboptimal maternal environment, such as poor diet, excess gestational weight gain, or gestational diabetes, increases offspring risk of obesity and metabolic syndrome in humans and in animal models [8,9]. In mouse and nonhuman primate (NHP) models, we and others have demonstrated that fetuses of high-fat diet (HFD)-fed dams show early signs of NAFLD, i.e., elevated liver triglycerides (TGs), oxidative stress, and evidence for periportal collagen deposition and stellate cell activation [10,11,12]. This lipotoxic phenotype persisted in NHP offspring through 1 year of age, even after implementing a healthy diet postweaning [12]. Moreover, switching NHP dams to a healthy diet prior to pregnancy prevented these effects in offspring liver [13]. In humans, maternal lipid concentrations, especially in early pregnancy, are associated with higher hepatic fat in adolescent offspring [14,15]. These findings demonstrate that maternal nutrition has long-lasting effects on offspring metabolic health and that fetal programming of hepatic lipid metabolism may be an early indicator of metabolic dysfunction in youth.

A growing body of evidence suggests that the species of lipids, their relative abundance, and their interplay are of central importance to the process of lipotoxicity. Sphingolipids, particularly sphingomyelins and ceramides, are structural components of cellular membranes and play a crucial role in cellular signaling events including cell division, differentiation, gene expression, immunomodulation, erythrocyte oxygen transport and offloading (critical to fatty acid (FA) oxidation capacity in mitochondria), and apoptosis [16,17,18,19]. For example, ceramides have been shown to inhibit insulin signaling and induce oxidative stress and inflammation [20]. Certain FA-ceramide species are pathogenic. Notably, ceramides with long FA chains such as CER 16:0 and CER 18:0 are involved in insulin resistance and hepatic steatosis [21,22]. However, very-long-chain (VLC) ceramides, such as CER 20:0, CER 22:0, and CER 24:0 [23,24,25,26], are protective. In addition, phosphatidylcholine (PC) and phosphatidylethanolamine (PE) are the most abundant phospholipids in all mammalian cell membranes and participate in epigenetic regulation and oxidant stress-induced protein isoaspartyl-damage repair by serving as methyl-group donors [27]. Studies have shown that both abnormally high and low molar ratios of PC/PE are linked to disease progression; specifically, increased hepatic PC/PE ratio has been linked to fatty liver disease [28]. Although the role of different lipid species in the pathogenesis of metabolic diseases is of considerable interest, our knowledge concerning specific lipid species with respect to mechanisms for developmental programming remains extremely limited. Moreover, therapeutic molecules delivered during gestation and lactation aimed at limiting accumulation of metabolically toxic lipid species throughout their lifespan are few and far between.

Pyrroloquinoline quinone (PQQ), an aromatic tricyclic o-quinone, is a dietary nutrient present in a variety of foods. PQQ is a potent antioxidant, protecting tissues from oxidative stress, decreasing levels of free radicals, and attenuating lipid peroxidation [29]. Studies in vivo and in vitro have shown that PQQ protects against oxidative damage and irradiation injury [30]. We have previously shown that maternal exposure to PQQ has lasting beneficial effects on hepatic lipid accumulation and PPARα signaling in offspring liver [31,32]. However, the effect of maternal diet and PQQ on pathways for hepatic lipid composition and disease progression has not been determined. In the present study, we employed untargeted lipidomics approaches to systematically identify changes in lipid composition in offspring livers from mothers fed a Western diet (WD) and supplemented with PQQ. This approach enables simultaneous measurements and relative quantification of a large number of lipid mediators. We hypothesized that maternal WD results in dysregulation of specific bioactive lipid metabolites that can be corrected by early exposure to PQQ to prevent NAFLD.

## 2. Results

### 2.1. PQQ Protects Offspring of Obese Dams from Weight Gain, Even When Only Exposed to PQQ during Gestation and Lactation

**Figure 1:**
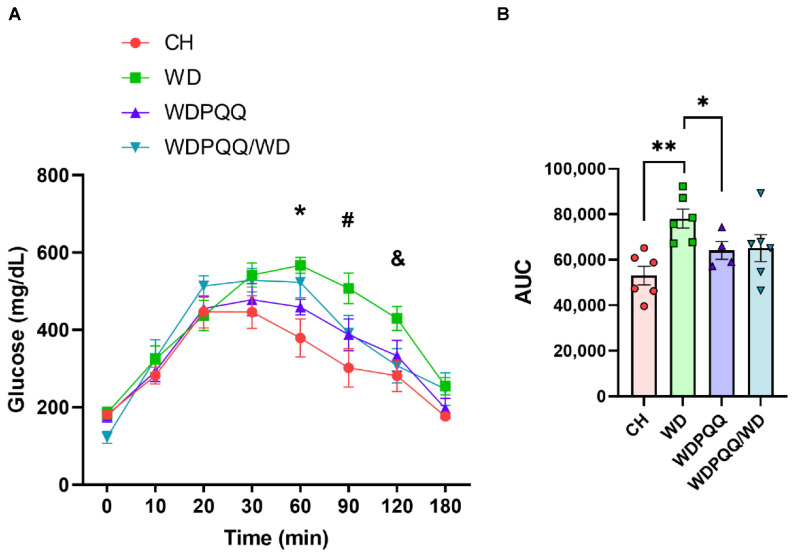
Chronic PQQ supplementation ameliorates WD-induced impaired glucose tolerance in 20 to 24 wk-old offspring. (**A**) Glucose tolerance test (GTT) measurements were obtained at baseline and 10, 20, 30, 60, 90, and 180 min following glucose injection. Data are means ± SEM, *n* = 4–6/group. * *p* < 0.01 CH and WDPQQ vs. WD; # *p* < 0.01 CH vs. WD; & *p* < 0.05 CH and WDPQQ/WD vs. WD using Student’s *t* test. (**B**) Area under the curve (AUC) from GTT. * *p* < 0.05, ** *p* < 0.01 vs. WD using Student’s *t* test. Data are means ± SEM, *n* = 4–6/group.

To determine the effect of acute and chronic PQQ to prevent developmental programing across the lifespan, PQQ was administered to WD-fed dams during gestation and lactation and offspring were weaned onto WD or WD with or without PQQ supplementation and studied at 20–24 wks of age. We studied four groups of adult offspring: offspring born to chow-fed dams and weaned onto maternal diet (CH); offspring born to WD-fed dams and weaned onto maternal diet (WD); offspring born to WD-fed, PQQ-supplemented dams and weaned onto WD with PQQ supplementation (chronic exposure to PQQ; WDPQQ); and offspring born to WD-fed dams with PQQ supplementation only during gestation and lactation and weaned onto WD without PQQ supplementation (maternal exposure to PQQ; WDPQQ/WD). We previously found that WD-induced maternal weight gain and serum TGs were attenuated by PQQ treatment [31]. In the current study, we found that maternal PQQ, given to dams during gestation and lactation only, protected adult offspring from maternal WD-induced weight gain, reduced liver hypertrophy, and improved fat/lean mass (Table 1). Moreover, chronic PQQ supplementation improved glucose tolerance in adult offspring (Figure 1).

### 2.2. PQQ Reduces Hepatic Steatosis, Beginning in Fetal Life, and Improves Lipid Metabolism in Adults

**Figure 2:**
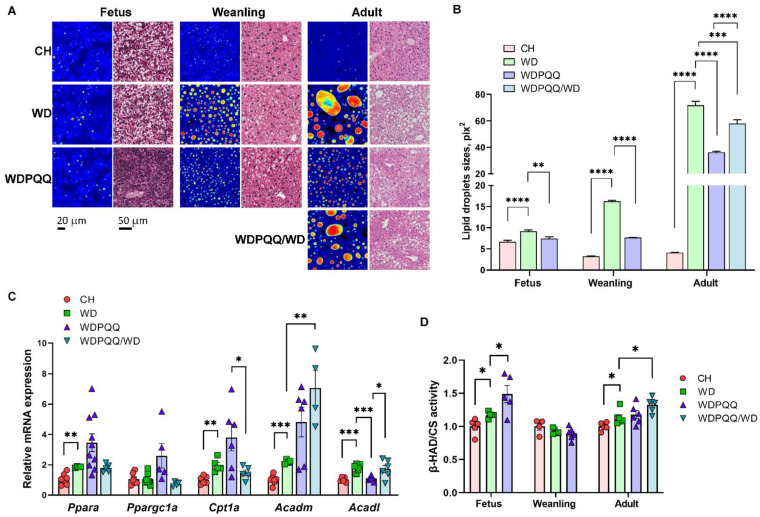
Hepatic steatosis in WD-fed offspring is improved by PQQ. (**A**) FLIM images and H&E staining of liver cryosections in fetuses (E18.5), weanlings (3 wks of age), and adults (20–24 wks of age). Scale bars: 20 μm for FLIM and 50 μm for H&E stains. Images acquired at 400× final magnification for FLIM and 40× for H&E. (**B**) Lipid droplet areas were quantified using ImageJ. Data are means ± SEM. ** *p* < 0.01, *** *p* < 0.001, **** *p* < 0.0001. (**C**) qPCR of genes involved in fatty acid oxidation in adult liver tissue. 18S was used as a reference gene. Data are means ± SEM. ** *p* < 0.01, *** *p* < 0.001. (**D**) Activity of β-HAD in liver homogenates. Citrate synthase (CS) activity was used for normalization. Data are means ± SEM. * *p* < 0.05 compared with WD.

Since hepatic lipid accumulation precedes hepatic insulin resistance [33], we hypothesized that changes in glucose tolerance result from altered hepatic lipid accumulation. Therefore, we employed histological analysis of fixed liver sections, as well as fluorescence lifetime imaging microscopy (FLIM) of fresh-frozen liver sections, to assess lipid accumulation in E18.5 fetuses, 3 wk-old weanlings, and 20 to 24 wk-old adult offspring (Figure 2A). Images from H&E staining of fixed liver sections and FLIM show a significant increase in lipid storage throughout the lifespan in offspring exposed to maternal WD (Figure 2A). We found a striking decrease in steatosis in PQQ-supplemented offspring, beginning in fetal liver and continuing through adulthood. Partial protection from fat accumulation was observed in offspring exposed to maternal PQQ only (WDPQQ/WD). Lipid droplet sizes quantified from the FLIM images were significantly decreased by both short-term maternal PQQ in fetuses, weanlings, and adults and by chronic, continuous exposure to PQQ in adults (Figure 2B). These findings are consistent with our previous report showing similar decreases in hepatic TGs in both groups of offspring exposed to PQQ [31].

To investigate potential mechanisms by which PQQ reduced hepatic fat accumulation, we used qPCR to measure mRNA expression levels of genes involved in mitochondrial FA oxidation (FAO) in livers from adult mice (Figure 2C). We found that expression levels of the PPARα cofactor *Ppargc1a* were increased in WDPQQ (*p* = 0.0504 vs. WD) and the downstream target *Acadm* was markedly increased in WDPQQ/WD (*p* = 0.006 vs. WD). Interestingly, while *Acadl* expression increased in WDPQQ and WDPQQ/WD, chronic exposure to PQQ (WDPQQ) increased expression of *Acadm* (*p* = 0.065 vs. WD) but not *Acadl*, suggesting that PQQ promotes accumulation of longer-chain FAs. In addition, chronic exposure to PQQ led to trend upregulation of the *Ppara* downstream target gene *Cpt1a* (*p* = 0.09), enhancing FA transfer into mitochondria for β-oxidation. We also measured the activity of 3-hydroxyacyl-CoA dehydrogenase (β-HAD)—the trifunctional protein that regulates β-oxidation of medium-chain FAs—and found that its activity increased in response to exposure to maternal PQQ in fetuses (*p* = 0.0417) and adults (*p* = 0.011), but not in response to chronic PQQ (Figure 2D).

### 2.3. Maternal PQQ Alters Lipid Profiles in WD-Fed Offspring

**Figure 3:**
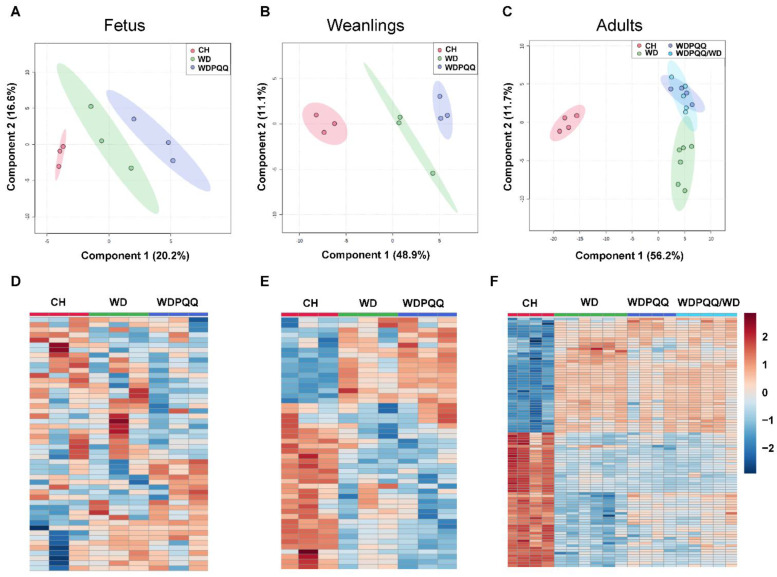
Diet and PQQ alter hepatic lipid profiles. MetaboAnalyst 5.0 was used to analyze mass-spectrometry-based lipidomics datasets from fetuses, weanlings, and adults. Data were normalized using log10 transformation and autoscaling. PLS-DA score plots for fetuses (**A**), weanlings (**B**), and adults (**C**). Heatmaps with significantly altered lipids for fetuses (**D**), weanlings (**E**), and adults (**F**).

Given the striking decrease in lipid droplet size in livers from PQQ-supplemented WD-fed offspring, we sought to determine whether overall lipid abundance or specific lipid classes were decreased in fetuses and offspring exposed to PQQ. We performed untargeted mass spectrometry-based lipidomics and observed that hepatic lipid species cluster differentially by exposure to maternal diet and PQQ throughout the lifespan. PLS-DA analysis was performed and score plots from fetuses, weanlings, and adults show that exposure to PQQ, whether given during the perinatal period only or chronically, altered the compositional distribution of lipids in the liver (Figure 3A–C). In fetuses and weanlings, lipid features clustered by diet and lipids from PQQ-exposed mice clustered separately. In adults, lipid features from PQQ-exposed mice clustered together, with little overlap with features from WD-fed mice (Figure 3C). Hierarchical clustering of the top 50 significantly altered lipid features for fetal and weanling livers (Figure 3D,E, respectively) and top 100 features for adult livers (Figure 3F) (*p* < 0.05 by ANOVA) showed that the effects of maternal diet are much more variable in fetal liver than in weanlings or adults.

**Figure 4:**
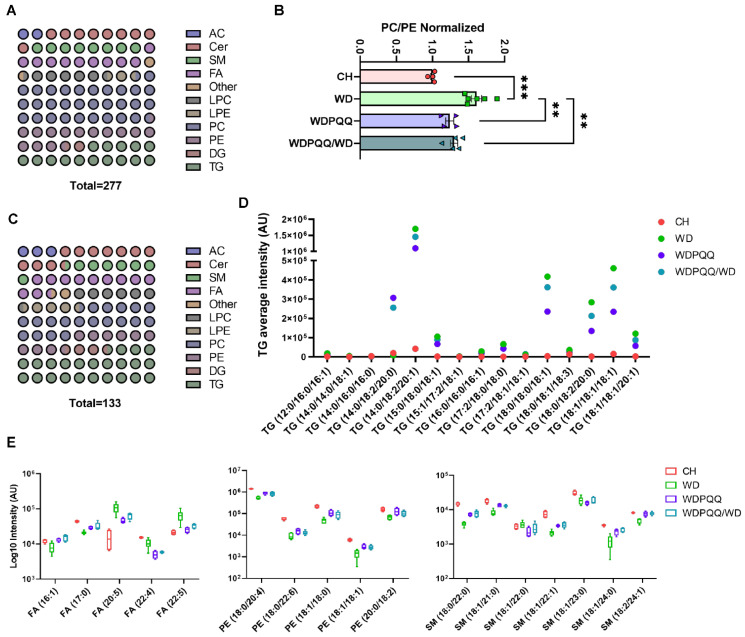
Exposure to PQQ alters the compositional distribution of lipids in adult liver. (**A**) Dot plot of the fractional distribution of total hepatic lipids in adult liver. (**B**) Ratio of phosphatidylcholines (PC) to phosphatidylethanolamine (PE). Data are means ± SEM. ** *p* < 0.01, *** *p* < 0.001. (**C**) Dot plot of the fractional distribution of significantly changed hepatic lipids in adult liver. (**D**) Scatter plot of average mass spectral intensities of the 15 core triglycerides. (**E**) Box plots of the log10 of the spectral intensity for lipid classes and chain lengths that were the most affected by PQQ (*n* = 4–6/group). AC, acylcarnitines; Cer, ceramides; DG, diacylglycerides; FA, fatty acids; LPC, lysophosphatidylcholine; LPE, lysophosphatidylethanolamine; PC, phosphatidylcholines; PE, phosphatidylethanolamines; SM, sphingomyelins; TG, triglycerides.

A limited mass-spectrometry-based analysis of lipids was performed in livers from fetuses and weanlings and a more comprehensive analysis was performed in adults. In fetus and weanling livers, 73 lipids were identified; a broader range of lipids (277) were identified in livers from adult offspring (Figure 4A). Lipid classes identified in adult livers were dominated by PC. The total PC/PE ratio was significantly elevated in WD-fed adult offspring and was rescued in offspring exposed to PQQ (Figure 4B). Dot plot analysis of lipids with significant differences between adult offspring groups showed that, considering all groups, predominant changes were in the PC, PE, and TG classes (Figure 4C), with the number of PC and PE species declining by over 60% and the number of TG species increasing by 57%.

Volcano plot analysis of the top 50 lipid features in fetal liver revealed seven significantly altered lipids when comparing CH vs. WD groups and two altered lipid features comparing WDPQQ vs. WD groups (Supplementary Table S1), suggesting that maternal diet and PQQ exposure have less effect on liver lipid profiles during gestation. In weanlings, the relative abundance of 28 lipids was significantly altered comparing CH vs. WD groups, whereas only two medium-chain acylcarnitines, hexanoyl-L-carnitine (AC (6:0)) and octenoyl-L-carnitine (AC (8:1)), were uniquely decreased in WDPQQ vs. WD groups (Supplementary Table S1). Changes in hepatic lipids in adult mice were identified at the species level using volcano plot analysis of the top 100 lipid features, which showed 121 significantly changed lipids in the CH vs. WD comparison, 39 lipids in the WDPQQ vs. WD comparison, and 21 lipids changed in the comparison of WDPQQ/WD vs. WD (Supplementary Table S2). Lipids that changed in CH vs. WD and in at least one other comparison group, or were altered only by maternal PQQ, were tabulated and 41 “core” lipids were identified (Supplementary Table S3). WD-fed mice showed an elevation of TGs compared with CH-fed mice and these TGs, particularly the most abundant, were significantly reduced with chronic PQQ exposure (Figure 4D). The effect of maternal PQQ was most notable for reducing the more highly abundant TGs, particularly TG (14:0/18:2/20:0), TG (14:0/18:2/20:1), and TG (18:1/18:1/18:1). The log10 relative abundance of the top lipid species changed by PQQ exposure was analyzed (Figure 4E). Welch’s *t* tests were performed, comparing each group with WD; the *p* values for each comparison are summarized in Table 2. PQQ exposure led to an exacerbated effect in several lipids, either further increasing (Cer (18:1/25:0)) or decreasing (Cer (18:0/18:1) and FA (22:4)) lipid abundance as compared with those in WD offspring liver. Hierarchical clustering shows enrichment of 29 lipids in adult liver, which cluster in both chronic and maternal PQQ-exposed mice (Supplementary Table S4). Of the core lipids identified in the volcano plot analyses in adults, six biolipids were also identified amongst the group of lipids affected by PQQ (Supplementary Table S4). Thus, for many core lipids, PQQ exposure partially ameliorated the effect of WD feeding, with chronic PQQ exerting a more robust effect.

**Figure 5:**
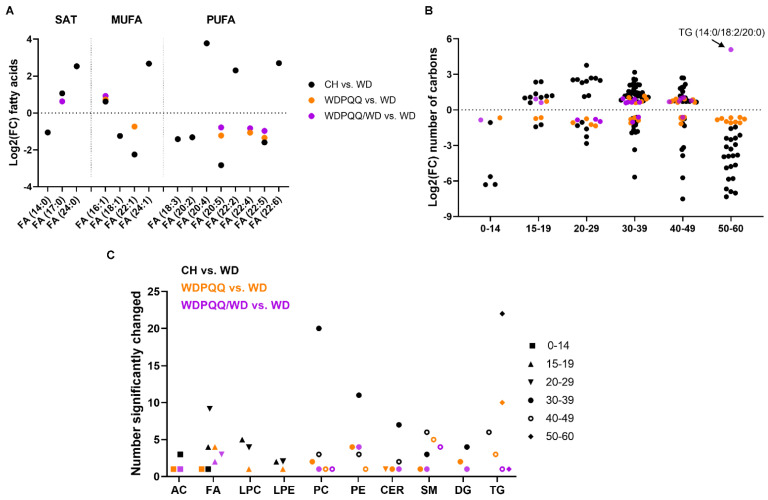
Diet and PQQ alter relative enrichment of double bonds and carbons in hepatic lipids from adult mice. Scatter plots of log2 fold change (FC) as a function of (**A**) fatty acids and (**B**) number of carbons. (**C**) Plot of the number of lipid features that were significantly changed in each lipid species with chain length indicated. Symbol colors indicate comparison groups, i.e., black = CH vs. WD, orange = WDPQQ vs. WD, and purple = WDPQQ/WD vs. WD. AC, acylcarnitines; CER, ceramides; DG, diacylglycerides; FA, fatty acids; LPC, lysophosphatidylcholine; LPE, lysophosphatidylethanolamine; MUFA, monounsaturated; PC, phosphatidylcholines; PE, phosphatidylethanolamines; PUFA, polyunsaturated fatty acids; SAT, saturated; SM, sphingomyelins; TG, triglycerides.

Given the shifts in lipid species and increases in genes for FAO in livers from adult offspring, we next sought to determine whether exposure to diet or PQQ alters fatty acyl chain lengths and saturation. Using significantly changing lipids from volcano plot analyses in adults, we plotted the log2 fold enrichment for all saturated FAs, monounsaturated FAs (MUFAs), and polyunsaturated FAs (PUFAs) (Figure 5A). More negative values of log2 fold changes reflect increased levels in WD-fed offspring compared with other diet groups. More positive values reflect increased levels in diet groups compared with those in WD. The relative differences in log2 fold change abundances between FAs (14:0) and (24:0) show that the abundance of saturated medium-chain FA was increased by WD whereas that of VLC FA was decreased, with little effect of PQQ exposure. On the other hand, PQQ rescued levels of MUFA (16:1) and chronic PQQ reduced levels of FA (22:1) due to WD. WD feeding strongly reduced levels of the PUFAs (20:4), (22:2), and (22:6) while PQQ treatment, and especially maternal PQQ, strikingly increased the log2 fold change of FA (20:5) compared with WD-fed offspring.

We next plotted the log2 fold enrichment values for different carbon chain lengths and found that lipids containing 30–38 carbons were less abundant in WD-fed mice and chronic PQQ partially rescued the lipid abundance (Figure 5B). Abundance of longer-chain lipids such as TGs were strongly increased by WD feeding (lower log2 fold change) and were improved by chronic PQQ. Again, abundance of TG (14:0/18:2/20:0) shows strong enrichment by maternal PQQ. Finally, the numbers of significantly changed lipid features were plotted as a function of lipid species and chain length (Figure 5C). As expected, most of the change in lipid abundance was found in the CH vs. WD comparison group, with phospholipids containing 30–38 carbons and long-chain TGs showing the greatest number of changing lipid species. Chronic PQQ predominantly affected enrichment of longer-chain sphingomyelins and PEs with 30–38 carbons, whereas maternal PQQ altered enrichment of more long-chain and VLC FAs, as well as sphingomyelins.

We also investigated the specific effect of maternal PQQ in WD-fed dams on lipid composition in WD-exposed offspring throughout the lifespan. Using volcano plot analyses, we tabulated the lipids with abundance significantly altered in livers from fetuses, weanlings, and adults exposed to maternal PQQ compared with unexposed counterparts (Table 3). Very few differences were noted between WD and WPQQ-exposed mice in fetal liver; however, we observed a modest increase in PUFA (FA (22:6)) abundance and ethanolamine phosphate—a product of sphingolipid metabolism—that paralleled the increase in abundance of several PUFAs and sphingomyelins in adult liver. Overall, these findings suggest that PQQ, given during pregnancy and lactation, blunts hepatic steatosis in offspring beginning in utero via modification of pathways involved in metabolism of PUFAs and sphingomyelins. Chronic PQQ predominantly affected the enrichment of longer-chain sphingomyelins and PEs with 30–38 carbons in WD-fed offspring, whereas maternal PQQ given only during gestation altered enrichment of more long-chain and VLC FAs and increased abundance of sphingomyelins in adult liver.

## 3. Discussion

Findings from studies in humans and animal models suggests that exposure to an obesogenic diet during the perinatal period increases an individual’s risk of developing adult metabolic diseases, including obesity and NAFLD, in childhood [11,12,14,34,35,36]. Emerging literature suggests that supplementation with antioxidants such as PQQ [32], N-acetylcysteine [37], and gut microbial products, including the short-chain FA butyrate [38,39,40], during gestation blunts the detrimental effects of maternal obesity on offspring metabolic health.

PQQ is a natural antioxidant found in soil, cruciferous vegetables, and enriched in human breast milk [41]. Here, we demonstrate that maternal supplementation with PQQ at a subpharmacological dose protected offspring from maternal WD-induced weight gain and developmental programming of NAFLD. Consistent with findings from earlier studies using diets depleted with PQQ [29,42,43], we used a diet-induced model of maternal obesity and demonstrated that the protective effects of PQQ on liver health began in utero—with no adverse effects on reproductive indices such as placental area [31], litter size, or offspring viability—and persisted into adulthood [32]. The present study expands on our previous findings and provides a comprehensive view of the changes in hepatic lipids that may contribute to the profound protection from NAFLD in PQQ-exposed offspring of obese pregnancy.

### 3.1. PQQ Improves Mitochondrial FAO

Hepatic steatosis is a hallmark of NAFLD and is considered the cause and the consequence of aberrations in liver lipid metabolism [44]. Our histological and FLIM data show extensive steatosis induced by maternal WD that was strikingly attenuated by PQQ. In NAFLD, hepatic lipid accumulation results from increased lipid uptake and de novo lipogenesis concomitant with impaired or insufficient mitochondrial FAO [45]. Subsequently, excess lipids promote cellular damage and disease progression by inducing oxidative stress, particularly when mitochondrial function is compromised [44]. Previous studies from our group and others have demonstrated a protective effect of PQQ against FA-induced oxidative stress and inflammation [31,46]. In the present study, we observed that maternal WD increased *Ppara* mRNA, which was further elevated in livers from chronically PQQ-exposed offspring. PPARα is a ligand-inducible transcription factor controlling expression of genes involved in breakdown of TGs, microsomal and peroxisomal lipid metabolism, and mitochondrial FAO [47]. Notably, maternal treatment with PQQ significantly increased activity of fetal and adult β-HAD, a mitochondrial trifunctional protein encoded by the gene *Hadhb* that catalyzes the last three steps in mitochondrial FAO. Although no change in β-HAD activity was observed in weanlings, the excess fat consumed during lactation is likely a key driver of mitochondrial FAO, prevailing over maternal diet effects. Our findings suggest that PQQ attenuates lipid accumulation in fetal and adult livers, in part, by upregulating mitochondrial FAO.

### 3.2. Long-Duration PQQ Exposure Alters Saturation of Hepatic Lipids

In human NASH, both saturated FAs and MUFAs accumulate in liver and serum [48,49]; animal models suggest that these lipids exacerbate NASH development [50]. In contrast, PUFAs exert beneficial effects on glucose and lipid metabolism, as well on the mitochondrial respiratory chain [51,52,53], and may delay the onset of NAFLD. Our findings show that the relative abundance of VLC FAs in offspring liver was decreased by WD. PQQ rescued this effect for MUFAs and PUFAs but had little impact on saturated FAs.

Of the PUFA species, levels of FA (20:5)/eicosapentaenoic acid (EPA; C20:5n − 3), an anti-inflammatory n − 3 PUFA enriched in fish oil [54], were elevated in PQQ-supplemented adult offspring whereas FA (22:4)/docosatetraenoic acid—an n − 6 PUFA—was reduced. EPA supplementation to mice fed an obesogenic diet improved metabolic parameters by reducing TG synthesis and promoting FAO [55,56]. Notably, in mice, increasing the n − 3:n − 6 FA ratio in dams attenuated developmental programming of NAFLD in maternal WD-exposed offspring [57]. Accordingly, elevated levels of protective PUFAs might contribute to improved mitochondrial function (i.e., increased β-HAD activity and PPARα activation) to attenuate development of metabolic disease in PQQ-supplemented offspring. Further, because PUFAs promote lipid oxidation and PPARα activation, resulting in increased energy expenditure [58], these findings suggest that a shift in the n − 3:n − 6 FA ratio due to PQQ might contribute to the attenuated weight gain observed in PQQ-supplemented offspring. In the future, it will be important to determine whether maternal PQQ alters offspring adipose tissue thermogenesis and energy balance.

### 3.3. PQQ Supplementation Elevates Abundance of VLC FAs in Liver

We next investigated effects of WD and PQQ on the distribution of carbon chain lengths in hepatic lipids. Consistent with observations in other studies demonstrating depletion of VLC FAs in obese patients [59,60,61], we found that VLC FAs with C30–C38 were depleted in livers from WD-fed offspring. Most saturated and monounsaturated VLC FAs are acyl moieties of sphingolipids, ceramides, and glucosylceramides [62,63]. Surprisingly, we observed elevated levels of VLC ceramides in PQQ-supplemented mice. These findings are consistent with a recent report using N-acetylcysteine supplementation to HFD-fed dams [37]. VLC ceramide deficiency has been linked to intestinal barrier permeability [26,64], an aggravating condition in obesity and NAFLD that results from gut bacterial dysbiosis. Our previous studies demonstrated an important role for PQQ in the protection against gut leakiness by increasing mRNA expression of *Tjp1* [32]. PQQ is an essential bacterial cofactor; however, little is known about the effects of PQQ on sphingolipid-producing bacteria. It is possible that the PQQ-mediated increase in VLC FAs results from either bacterial or host production of bioactive sphingolipids in the gut, which is a question for future investigation.

### 3.4. PQQ Alters Abundance of Short-Chain Acylcarnitines and Long-Chain Glycerolipids in Early Life

Acylcarnitines are FA intermediate metabolites formed during FAO, and their accumulation is associated with insulin resistance and NAFLD [65,66]. Indeed, acylcarnitines have been proposed as biomarkers for screening NAFLD due to the positive correlation between their accumulation and NAFLD progression [65]. Here, we observed that levels of short-chain acylcarnitines AC (3:0) and AC (4:0) were reduced in livers from PQQ-exposed weanlings compared with non-PQQ-exposed counterparts, concomitant with decreased steatosis. Inhibiting shorter-chain acylcarnitine formation in mouse or human primary myotubes was found to protect against palmitate-induced insulin resistance and reactive oxygen species production [67]. Thus, one beneficial effect of PQQ on reducing oxidative stress likely stems from reducing acylcarnitines concomitant with increases in hepatic FAO.

Glycerolipids, including DGs and TGs, constitute over 99% of lipid species and play important roles in NAFLD development, inducing oxidative stress and lipotoxicity [68]. A previous study showed that a PQQ-deficient diet resulted in elevated TGs, whilst replenishment with PQQ reduced TGs to protect against ischemia/reperfusion-induced cardiac injury [69]. In the present study, our analysis of TG distributions revealed that maternal WD increases TGs in offspring and PQQ exposure rescued this increase, with the main difference arising from decreases in VLC TGs (TG (14:0/18:2/20:1), TG (18:0/18:0/18:1), TG (18:0/18:2/20:0), and TG (18:1/18:1/18:1)). A reduction in VLC TGs may thus contribute to the enhanced FAO we observed in PQQ-exposed offspring. Hepatic TG levels are driven by de novo lipogenesis, which is regulated by the transcription factors SREBP-1c and ChREBP-1 [70,71]. Several lines of evidence indicate that activation of AMPK–SIRT1 signaling pathway plays a major role in the suppression of lipogenesis and leads to a subsequent reduction in TG levels in HFD-fed mice [72]. PQQ promotes production of NAD+ and increases SIRT1 activity in hepatocytes [73] and animal tissues [74,75,76]. Further, a recent study demonstrated that PQQ supplementation inhibited the nuclear translocation and activity of SREBP-1c via AMPK activation, which led to a reduction in lipogenesis in HFD-fed mice [77].

### 3.5. PQQ Supplementation Attenuates WD-Induced Increase in PC/PE

In mammals, phosphatidylcholines and phosphatidylethanolamines (PC and PE, respectively) are the most abundant phospholipids in all cell and mitochondrial membranes. These lipids are increasingly recognized as important molecules for metabolic control [28]. Elevated PC and reduced PE levels, increasing the PC/PE ratio, lead to ER stress by inhibiting sarco/endoplasmic reticulum calcium ATPase (SERCA) activity; reducing the PC/PE ratio is beneficial for glucose metabolism [78]. Here, we found that the total PC/PE ratio was elevated in WD-fed offspring and was partially rescued by both maternal and chronic PQQ. We ascribe this change primarily to elevated levels of PE such as PE (18:0/20:4), PE (18:0/22:6), PE (18:1/18:0), and PE (18:1/18:1) in the livers of PQQ-exposed offspring. PE (18:0/20:4), a phospholipid originating in the placenta, was the most abundant of the significantly changing lipids in the liver. Interestingly, a study in pregnant women revealed a positive correlation of PE (18:0/20:4) abundance with fetal docosahexaenoic acid, as well as both placental and infant growth [79], suggesting that further studies are warranted on mechanisms for fetal growth using PQQ.

In summary, maternal PQQ, starting in early gestation, provides long-term protection from WD-induced NAFLD in the offspring. Our findings suggest that the mechanisms begin in utero and are pleiotropic, including increasing the abundance of VLC ceramides, reducing the hepatic PC/PE ratio, and increasing the ratio of protective n − 3 to n − 6 PUFAs. PQQ increased hepatic FAO genes and protected WD-fed offspring from excess weight gain while improving glucose tolerance and reducing fat mass. Importantly, oxidative stress is a key mechanism contributing to pathophysiology in NAFLD. PQQ’s antioxidant activity has been extensively reported and likely contributes to the observed decline in TG accumulation by reduction of reactive oxygen species and oxidation of free fatty acids [80]. A limitation of this study is that postnatal WD feeding and PQQ supplementation may obscure the molecular changes driven only by maternal exposures. Therefore, future work will expand upon these initial findings using a model in which all pups are weaned to a control diet. Future studies investigating these effects on adipose tissue energy metabolism and gut function, as well as epigenetic modifications in the offspring, will also be important. Given the fact that PQQ is a natural nutritional substance that has been used safely as a supplement to blunt the effects of cognitive decline in human aging, cholesterol, and dry skin, it may be conceivable to test PQQ in pregnancy in higher species (primates, humans) for its impact on limiting developmental programming in the near future.

## 4. Materials and Methods

### 4.1. Animals and Diets

Six- to eight-week old C57BL/6J female mice were randomly grouped and fed either standard chow (CH; 2019; Envigo, Indianapolis, IN, USA; 22% kcal from fat, 23% protein, 55% carbohydrate, 3.3 kcal/g) or Western-style diet (WD; TD.88137; Envigo; 42% kcal from fat, 15% protein, 43% carbohydrate (34% sucrose by weight), 0.2% cholesterol, 4.5 kcal/g) ad libitum without and with Bio-PQQ (WDPQQ) in their drinking water (3.8 µM, as described in [31]). After 2 weeks, a CH-fed male was added to make a breeding pair and the pair continued with the female diet and drinking water throughout pregnancy. For adult offspring groups, males were weaned onto maternal diet (CH, WD, or WDPQQ) and continued until 20–24 weeks of age. A fourth set of male offspring were weaned onto WD without PQQ supplementation (WDPQQ/WD). For longitudinal analyses, groups of mice at gestation day E18.5 (fetal), 3 wk of age (weanling), or 20–24 wks of age (adult), were euthanized and their tissues dissected, weighed, snap frozen, and stored at −80 °C. Body composition was determined in adult male mice by quantitative magnetic resonance imaging (qMRI; Echo MRI Whole Body Composition Analyzer; Echo Medical Systems, Houston, TX, USA). Body composition was measured either before acclimation to the calorimeter or 24–48 h before death.

Livers were fixed in 4% paraformaldehyde overnight, embedded in paraffin, and cut onto glass slides as 5-μm sections. Sections were deparaffinized, rehydrated, and stained with H&E. Staining was visualized using an Aperio whole slide scanner (Leica Biosystems, Deer Park, IL, USA) and images were examined using ImageScope software (Leica). For GTTs, adult offspring were fasted for 4–5 h then injected i.p. with glucose (2 g/kg body weight). Blood glucose was measured with a glucometer by nicking the tail vein.

### 4.2. Fluorescence Lifetime Imaging Microscopy (FLIM)

FLIM was performed on liver cryosections to detect lipid droplets using a Zeiss 780 laser-scanning confocal/multiphoton-excitation fluorescence microscope with a 34-Channel GaAsP QUASAR Detection Unit and non-descanned detectors for 2-photon fluorescence (Zeiss, Thornwood, NY, USA) equipped with an ISS A320 FastFLIM box and a titanium:sapphire Chameleon Ultra II (Coherent, Santa Clara, CA, USA) as previously described [81]. Fifteen fields-of-view were taken for each sample; lipid droplet signal was thresholded in ImageJ2 (imageJ.net) and lipid droplet size was quantified using the Analyze Particles tool.

### 4.3. Lipidomics

Reverse-phased liquid chromatography tandem mass spectrometry (RPLC-MS/MS) was used to perform lipidomic analysis on extracts from frozen aliquots of liver tissue from adult mice at West Coast Metabolomics Center, as previously described [82]. Chromatography was performed using a Vanquish Focus UHPLC system (Thermo Fisher Scientific, Waltham, MA, USA) and mass spectra collected with an Agilent 6530 QTOF mass spectrometer (Agilent, Santa Clara, CA, USA) [83]. LC–MS/MS data were processed using open source software MS-DIAL [84], which performed peak picking, deisotoping, automated peak annotation, alignment, and gap filling. Blank subtraction was performed by removing features that had a maximum sample intensity/average blank intensity ratio of less than 5 and also any features that had a maximum sample intensity of less than 1K. Adduct and duplicate features were flagged using Mass Spectral Feature List Optimizer (MS-FLO) [85]. Peak height was used for all quantitation. Tandem MS/MS libraries of the MassBank of North America (MassBank.us) and NIST17 (NIST, Gaithersberg, MD, USA) were used for spectral matching.

Fetal and weanling liver samples were extracted in ice-cold methanol, prior to dilution 1:1 (*v*/*v*) with 10 mM ammonium acetate for analysis by UHPLC-MS at the CU Anschutz Metabolomics Core. The analytical platform employed a Vanquish UHPLC system coupled online to a QExactive mass spectrometer. Lipidomics analyses were performed as described previously [86,87]. Lipid assignments were performed using LipidSearch (Thermo Fisher); spectral libraries generated in LipidSearch for this dataset were then imported into Compound Discoverer (Thermo Fisher). Spectral libraries were generated from high-resolution accurate intact mass, isotopic pattern, and MS2 fragmentation spectra against the LipidMaps databases.

## Figures and Tables

**Table 1:** Effect of WD and PQQ administration on 20 to 24 wk-old adult offspring.

	CH	WD	WDPQQ	WDPQQ/WD	*p* Values		
	*n* = 6	*n* = 8	*n* = 7	*n* = 6	Interaction	Diet	PQQ
Age (wks)	23.1 ± 0.6	23.3 ± 1.4	21.5 ± 0.4	20.4 ± 0.03	0.113	0.176	0.952
Body weight (g)	27.7 ± 1.4	43.1 ± 0.8	34.3 ± 1.0	33.4 ± 0.5 ^1^	0.0023	<0.0001	<0.0001
Liver mass (% of BW)	4.5 ± 0.2	6.7 ± 0.5	4.7 ± 0.1	4.4 ± 0.4 ^1^	0.0004	0.0145	0.0952
Fat mass (% of BW)	17.2 ± 1.8	41.3 ± 1.0	34.6 ± 2.0	32.1 ± 1.4 ^1^	0.209	<0.0001	<0.0001
Lean mass (% of BW)	70.2 ± 2.2	53.0 ± 1.1	59.1 ± 2.0	57.0 ± 1.6 ^2^	0.345	<0.0001	0.0126

Data are means ± SEM. One-way ANOVA with multiple comparisons is shown. ^1^ *p* < 0.05 compared with WD offspring. ^2^ *p* = 0.053 compared with WD offspring. BW, body weight.

**Table 2:** *t*-tests with Welch’s correction of top lipids affected by PQQ in adult offspring.

	*p* Value		
Lipid	CH vs. WD	WDPQQ vs. WD	WDPQQ/WD vs. WD
FA (16:1)	**0.0479**	0.0660	0.0593
FA (17:0)	**0.0007**	**0.0394**	0.2085
FA (20:5)	**0.0093**	0.0784	0.1813
FA (22:4)	**0.0479**	0.0784	0.1499
FA (22:5)	**0.0479**	0.0920	0.1813
PE (18:0/20.4)	**<0.0001**	0.0784	0.1264
PE (18:0/22.6)	**0.0093**	0.2212	0.2322
PE (18:1/18.0)	**0.0168**	0.1334	0.2187
PE (18:1/18.1)	**0.0089**	0.1002	0.0866
PE (20:0/18.2)	**0.0479**	0.2213	0.2322
SM (18:0/22.0)	**0.0061**	**0.0089**	0.0607
SM (18:1/21.0)	**0.0168**	**0.0394**	**0.005**
SM (18:1/22.0)	0.3699	0.1181	0.3669
SM (18:1/22.1)	**0.0479**	**0.004**	0.0574
SM (18:1/23.0)	**0.0479**	0.2388	0.988
SM (18:1/24.0)	**0.0005**	0.1002	**0.0209**
SM (18:2/24.1)	**0.0005**	0.1002	**0.0054**

**Table 3:** Lipids affected by maternal PQQ throughout the lifespan.

WDPQQ vs. WD	Log2(FC)	−Log10(P)
Fetus		
FA (22:6)	0.6343	1.5199
ethanolamine phosphate	0.6512	2.2746
Weanling		
AC (6:0)	−1.7929	1.9059
AC (8:1)	−1.3661	1.6884
Adult		
AC (3:0)	−0.8425	1.1616
Cer (18:0/18:1)	−1.0216	1.8328
DG (18:1/18:1)	−0.6062	1.4309
FA (16:1)	0.9205	2.2385
FA (17:0)	0.6232	2.4215
FA (20:5)	−0.7813	1.637
FA (22:4)	−0.8306	2.0879
FA (22:5)	−0.9707	1.8923
PC (16:0/16.0)	0.6412	2.8177
PC (17:0/18.2)	0.7342	1.1069
PE (18:0/20.4)	0.643	2.5834
PE (18:1/18.0)	0.8959	1.8718
PE (18:1/18.1)	0.9549	1.4628
PE (20:4/18.2)	0.5958	3.1917
SM (18:0/22.0)	0.9733	3.6305
SM (18:1/21.0)	0.6561	3.2631
SM (18:1/22.1)	0.7783	3.1681
SM (18:1/24.0)	1.0566	1.8493
SM (18:2/24.1)	0.6891	3.3378
TG (12:0/16:0/16.1)	−0.612	1.5556
TG (14:1/18:2/20:0)	5.0953	1.5114

## Data Availability

Data were uploaded to the Metabolomics Workbench (https://www.metabolomicsworkbench.org, accessed on 18 May 2022). Data Track IDs 3208 (adults), 3209 (fetuses), and 3210 (weanlings). mwTab Filenames krjonscher_20_220420_084738, _101721, and _103447, respectively.

## Supplementary Materials

The following supporting information can be downloaded at: https://www.mdpi.com/article/10.3390/ijms23116043/s1.

## References

[1] Doycheva I., Watt K.D., Alkhouri N. (2017). Nonalcoholic fatty liver disease in adolescents and young adults: The next frontier in the epidemic. Hepatology.

[2] Younossi Z.M., Koenig A.B., Abdelatif D., Fazel Y., Henry L., Wymer M. (2016). Global epidemiology of nonalcoholic fatty liver disease-Meta-analytic assessment of prevalence, incidence, and outcomes. Hepatology.

[3] Matteoni C.A., Younossi Z.M., Gramlich T., Boparai N., Liu Y.C., McCullough A.J. (1999). Nonalcoholic fatty liver disease: A spectrum of clinical and pathological severity. Gastroenterology.

[4] Wesolowski S.R., El Kasmi K.C., Jonscher K.R., Friedman J.E. (2017). Developmental origins of NAFLD: A womb with a clue. Nat. Rev. Gastroenterol. Hepatol..

[5] Mandala A., Janssen R.C., Palle S., Short K.R., Friedman J.E. (2020). Pediatric non-alcoholic fatty liver disease: Nutritional origins and potential molecular mechanisms. Nutrients.

[6] Schwimmer J.B., Behling C., Newbury R., Deutsch R., Nievergelt C., Schork N.J., Lavine J.E. (2005). Histopathology of pediatric nonalcoholic fatty liver disease. Hepatology.

[7] Vajro P., Lenta S., Socha P., Dhawan A., McKiernan P., Baumann U., Durmaz O., Lacaille F., McLin V., Nobili V. (2012). Diagnosis of nonalcoholic fatty liver disease in children and adolescents: Position paper of the ESPGHAN Hepatology Committee. J. Pediatr. Gastroenterol. Nutr..

[8] Boney C.M., Verma A., Tucker R., Vohr B.R. (2005). Metabolic syndrome in childhood: Association with birth weight, maternal obesity, and gestational diabetes mellitus. Pediatrics.

[9] Schack-Nielsen L., Michaelsen K.F., Gamborg M., Mortensen E.L., Sørensen T.I. (2010). Gestational weight gain in relation to offspring body mass index and obesity from infancy through adulthood. Int. J. Obes. (Lond.).

[10] Oben J.A., Mouralidarane A., Samuelsson A.M., Matthews P.J., Morgan M.L., McKee C., Soeda J., Fernandez-Twinn D.S., Martin-Gronert M.S., Ozanne S.E. (2010). Maternal obesity during pregnancy and lactation programs the development of offspring non-alcoholic fatty liver disease in mice. J. Hepatol..

[11] McCurdy C.E., Bishop J.M., Williams S.M., Grayson B.E., Smith M.S., Friedman J.E., Grove K.L. (2009). Maternal high-fat diet triggers lipotoxicity in the fetal livers of nonhuman primates. J. Clin. Investig..

[12] Nash M.J., Dobrinskikh E., Newsom S.A., Messaoudi I., Janssen R.C., Aagaard K.M., McCurdy C.E., Gannon M., Kievit P., Friedman J.E. (2021). Maternal Western diet exposure increases periportal fibrosis beginning in utero in nonhuman primate offspring. JCI Insight.

[13] Wesolowski S.R., Mulligan C.M., Janssen R.C., Baker P.R., Bergman B.C., D’Alessandro A., Nemkov T., Maclean K.N., Jiang H., Dean T.A. (2018). Switching obese mothers to a healthy diet improves fetal hypoxemia, hepatic metabolites, and lipotoxicity in non-human primates. Mol. Metab..

[14] Cohen C.C., Francis E.C., Perng W., Sauder K.A., Scherzinger A., Sundaram S.S., Shankar K., Dabelea D. (2022). Exposure to maternal fuels during pregnancy and offspring hepatic fat in early childhood: The healthy start study. Pediatr. Obes..

[15] Cohen C.C., Perng W., Sauder K.A., Ringham B.M., Bellatorre A., Scherzinger A., Stanislawski M.A., Lange L.A., Shankar K., Dabelea D. (2021). Associations of nutrient intake changes during childhood with adolescent hepatic fat: The Exploring Perinatal Outcomes among Children Study. J. Pediatr..

[16] Marí M., Fernández-Checa J.C. (2007). Sphingolipid signalling and liver diseases. Liver Int..

[17] Garris C.S., Blaho V.A., Hla T., Han M.H. (2014). Sphingosine-1-phosphate receptor 1 signalling in T cells: Trafficking and beyond. Immunology.

[18] Sun K., Zhang Y., D’Alessandro A., Nemkov T., Song A., Wu H., Liu H., Adebiyi M., Huang A., Wen Y.E. (2016). Sphingosine-1-phosphate promotes erythrocyte glycolysis and oxygen release for adaptation to high-altitude hypoxia. Nat. Commun..

[19] Johnson D.R., Decker E.A. (2015). The role of oxygen in lipid oxidation reactions: A review. Annu. Rev. Food Sci. Technol..

[20] Chavez J.A., Summers S.A. (2012). A ceramide-centric view of insulin resistance. Cell Metab..

[21] Turpin S.M., Nicholls H.T., Willmes D.M., Mourier A., Brodesser S., Wunderlich C.M., Mauer J., Xu E., Hammerschmidt P., Brönneke H.S. (2014). Obesity-induced CerS6-dependent C16:0 ceramide production promotes weight gain and glucose intolerance. Cell Metab..

[22] Luukkonen P.K., Zhou Y., Sädevirta S., Leivonen M., Arola J., Orešič M., Hyötyläinen T., Yki-Järvinen H. (2016). Hepatic ceramides dissociate steatosis and insulin resistance in patients with non-alcoholic fatty liver disease. J. Hepatol..

[23] Kim Y.R., Lee E.J., Shin K.O., Kim M.H., Pewzner-Jung Y., Lee Y.M., Park J.W., Futerman A.H., Park W.-J. (2019). Hepatic triglyceride accumulation via endoplasmic reticulum stress-induced SREBP-1 activation is regulated by ceramide synthases. Exp. Mol. Med..

[24] Park W.J., Park J.W., Merrill A.H., Storch J., Pewzner-Jung Y., Futerman A.H. (2014). Hepatic fatty acid uptake is regulated by the sphingolipid acyl chain length. Biochim. Biophys. Acta.

[25] Saroha A., Pewzner-Jung Y., Ferreira N.S., Sharma P., Jouan Y., Kelly S.L., Feldmesser E., Merrill A.H., Trottein F., Paget C. (2017). Critical role for very-long chain sphingolipids in invariant natural killer T cell development and homeostasis. Front. Immunol..

[26] Kim Y.R., Volpert G., Shin K.O., Kim S.Y., Shin S.H., Lee Y., Sung S.H., Lee Y.M., Ahn J.-H., Pewzner-Jung Y. (2017). Ablation of ceramide synthase 2 exacerbates dextran sodium sulphate-induced colitis in mice due to increased intestinal permeability. J. Cell Mol. Med..

[27] D’Alessandro A., Hay A., Dzieciatkowska M., Brown B.C., Morrison E.J., Hansen K.C., Zimring J.C. (2021). Protein-L-isoaspartate O-methyltransferase is required for *in vivo* control of oxidative damage in red blood cells. Haematologica.

[28] van der Veen J.N., Kennelly J.P., Wan S., Vance J.E., Vance D.E., Jacobs R.L. (2017). The critical role of phosphatidylcholine and phosphatidylethanolamine metabolism in health and disease. Biochim. Biophys. Acta Biomembr..

[29] Smidt C.R., Steinberg F.M., Rucker R.B. (1991). Physiologic importance of pyrroloquinoline quinone. Proc. Soc. Exp. Biol. Med..

[30] Ouchi A., Nakano M., Nagaoka S., Mukai K. (2009). Kinetic study of the antioxidant activity of pyrroloquinolinequinol (PQQH(2), a reduced form of pyrroloquinolinequinone) in micellar solution. J. Agric. Food Chem..

[31] Jonscher K.R., Stewart M.S., Alfonso-Garcia A., DeFelice B.C., Wang X.X., Luo Y., Levi M., Heerwagen M.J., Janssen R.C., de la Houssaye B.A. (2017). Early PQQ supplementation has persistent long-term protective effects on developmental programming of hepatic lipotoxicity and inflammation in obese mice. FASEB J..

[32] Friedman J.E., Dobrinskikh E., Alfonso-Garcia A., Fast A., Janssen R.C., Soderborg T.K., Anderson A.L., Reisz J.A., D’Alessandro A., Frank D.N. (2018). Pyrroloquinoline quinone prevents developmental programming of microbial dysbiosis and macrophage polarization to attenuate liver fibrosis in offspring of obese mice. Hepatol. Commun..

[33] Jelenik T., Kaul K., Séquaris G., Flögel U., Phielix E., Kotzka J., Knebel B., Fahlbusch P., Hörbelt T., Lehr S. (2017). Mechanisms of insulin resistance in primary and secondary nonalcoholic fatty liver. Diabetes.

[34] Srinivasan M., Katewa S.D., Palaniyappan A., Pandya J.D., Patel M.S. (2006). Maternal high-fat diet consumption results in fetal malprogramming predisposing to the onset of metabolic syndrome-like phenotype in adulthood. Am. J. Physiol. Endocrinol. Metab..

[35] Desai M., Jellyman J.K., Han G., Beall M., Lane R.H., Ross M.G. (2014). Maternal obesity and high-fat diet program offspring metabolic syndrome. Am. J. Obs. Gynecol..

[36] Brumbaugh D.E., Friedman J.E. (2014). Developmental origins of nonalcoholic fatty liver disease. Pediatr. Res..

[37] Charron M.J., Williams L., Seki Y., Du X.Q., Chaurasia B., Saghatelian A., Summers S.A., Katz E.B., Vuguin P.M., Reznik S.E. (2020). Antioxidant effects of N-acetylcysteine prevent programmed metabolic disease in mice. Diabetes.

[38] Huang Y., Gao S., Jun G., Zhao R., Yang X. (2017). Supplementing the maternal diet of rats with butyrate enhances mitochondrial biogenesis in the skeletal muscles of weaned offspring. Br. J. Nutr..

[39] Lu H., Su S., Ajuwon K.M. (2012). Butyrate supplementation to gestating sows and piglets induces muscle and adipose tissue oxidative genes and improves growth performance. J. Anim. Sci..

[40] Lin Y., Fang Z.F., Che L.Q., Xu S.Y., Wu D., Wu C.M., Wu X.Q. (2014). Use of sodium butyrate as an alternative to dietary fiber: Effects on the embryonic development and anti-oxidative capacity of rats. PLoS ONE.

[41] Jonscher K.R., Rucker R.B., Watson R.R., Preedy V.R. (2019). Chapter 13—Pyrroloquinoline quinone: Its profile, effects on the liver and implications for health and disease prevention. Dietary Interventions in Liver Disease.

[42] Steinberg F.M., Gershwin M.E., Rucker R.B. (1994). Dietary pyrroloquinoline quinone: Growth and immune response in BALB/c mice. J. Nutr..

[43] Steinberg F., Stites T.E., Anderson P., Storms D., Chan I., Eghbali S., Rucker R. (2003). Pyrroloquinoline quinone improves growth and reproductive performance in mice fed chemically defined diets. Exp. Biol. Med. (Maywood).

[44] Ipsen D.H., Lykkesfeldt J., Tveden-Nyborg P. (2018). Molecular mechanisms of hepatic lipid accumulation in non-alcoholic fatty liver disease. Cell Mol. Life Sci..

[45] Loomba R., Friedman S.L., Shulman G.I. (2021). Mechanisms and disease consequences of nonalcoholic fatty liver disease. Cell.

[46] Wang Z., Li Y., Wang Y., Zhao K., Chi Y., Wang B. (2019). Pyrroloquinoline quinine protects HK-2 cells against high glucose-induced oxidative stress and apoptosis through Sirt3 and PI3K/Akt/FoxO3a signaling pathway. Biochem. Biophys. Res. Commun..

[47] Kersten S., Stienstra R. (2017). The role and regulation of the peroxisome proliferator activated receptor alpha in human liver. Biochimie.

[48] Puri P., Baillie R.A., Wiest M.M., Mirshahi F., Choudhury J., Cheung O., Sargeant C., Contos M.J., Sanyal A.J. (2007). A lipidomic analysis of nonalcoholic fatty liver disease. Hepatology.

[49] de Almeida I.T., Cortez-Pinto H., Fidalgo G., Rodrigues D., Camilo M.E. (2002). Plasma total and free fatty acids composition in human non-alcoholic steatohepatitis. Clin. Nutr..

[50] Larter C.Z., Yeh M.M., Haigh W.G., Williams J., Brown S., Bell-Anderson K.S., Lee S.P., Farrell G.C. (2008). Hepatic free fatty acids accumulate in experimental steatohepatitis: Role of adaptive pathways. J. Hepatol..

[51] Burri L., Berge K., Wibrand K., Berge R.K., Barger J.L. (2011). Differential effects of krill oil and fish oil on the hepatic transcriptome in mice. Front. Genet..

[52] Ferramosca A., Zara V. (2014). Modulation of hepatic steatosis by dietary fatty acids. World J. Gastroenterol..

[53] Ren C., Hou L., Liu B., Yang G.P., Wang Y.Y., Shi Q.Z. (2011). Distinct structures of coordination polymers incorporating flexible triazole-based ligand: Topological diversities, crystal structures and property studies. Dalton Trans..

[54] Albracht-Schulte K., Kalupahana N.S., Ramalingam L., Wang S., Rahman S.M., Robert-McComb J., Moustaid-Moussa N. (2018). Omega-3 fatty acids in obesity and metabolic syndrome: A mechanistic update. J. Nutr. Biochem..

[55] Albracht-Schulte K., Gonzalez S., Jackson A., Wilson S., Ramalingam L., Kalupahana N.S., Moustaid-Moussa N. (2019). Eicosapentaenoic acid improves hepatic metabolism and reduces inflammation independent of obesity in high-fat-fed mice and in HepG2 cells. Nutrients.

[56] Kalupahana N.S., Claycombe K., Newman S.J., Stewart T., Siriwardhana N., Matthan N., Lichtenstein A.H., Moustaid-Moussa N. (2010). Eicosapentaenoic acid prevents and reverses insulin resistance in high-fat diet-induced obese mice via modulation of adipose tissue inflammation. J. Nutr..

[57] Heerwagen M.J., Stewart M.S., de la Houssaye B.A., Janssen R.C., Friedman J.E. (2013). Transgenic increase in n-3/n-6 fatty acid ratio reduces maternal obesity-associated inflammation and limits adverse developmental programming in mice. PLoS ONE.

[58] Clarke S.D. (2001). Nonalcoholic steatosis and steatohepatitis. I. Molecular mechanism for polyunsaturated fatty acid regulation of gene transcription. Am. J. Physiol. Gastrointest. Liver Physiol..

[59] Fox T.E., Bewley M.C., Unrath K.A., Pedersen M.M., Anderson R.E., Jung D.Y., Jefferson L.S., Kim J.K., Bronson S.K., Flanagan J.M. (2011). Circulating sphingolipid biomarkers in models of type 1 diabetes. J. Lipid Res..

[60] Oda E., Hatada K., Kimura J., Aizawa Y., Thanikachalam P.V., Watanabe K. (2005). Relationships between serum unsaturated fatty acids and coronary risk factors: Negative relations between nervonic acid and obesity-related risk factors. Int. Heart J..

[61] Yamazaki Y., Kondo K., Maeba R., Nishimukai M., Nezu T., Hara H. (2014). Proportion of nervonic acid in serum lipids is associated with serum plasmalogen levels and metabolic syndrome. J. Oleo Sci..

[62] Uchida Y. (2011). The role of fatty acid elongation in epidermal structure and function. Dermato-Endocrinology.

[63] Kihara A. (2012). Very long-chain fatty acids: Elongation, physiology and related disorders. J. Biochem..

[64] Oertel S., Scholich K., Weigert A., Thomas D., Schmetzer J., Trautmann S., Wegner M.S., Radeke H.H., Filmann N., Brüne B. (2017). Ceramide synthase 2 deficiency aggravates AOM-DSS-induced colitis in mice: Role of colon barrier integrity. Cell Mol. Life Sci..

[65] Chang Y., Gao X.Q., Shen N., He J., Fan X., Chen K., Lin X.H., Li H.M., Tian F.-S., Li H. (2020). A targeted metabolomic profiling of plasma acylcarnitines in nonalcoholic fatty liver disease. Eur Rev. Med. Pharmacol. Sci..

[66] Mihalik S.J., Goodpaster B.H., Kelley D.E., Chace D.H., Vockley J., Toledo F.G., DeLany J.P. (2010). Increased levels of plasma acylcarnitines in obesity and type 2 diabetes and identification of a marker of glucolipotoxicity. Obesity (Silver Spring).

[67] Aguer C., McCoin C.S., Knotts T.A., Thrush A.B., Ono-Moore K., McPherson R., Dent R., Hwang D.H., Adams S.H., Harper M.E. (2015). Acylcarnitines: Potential implications for skeletal muscle insulin resistance. FASEB J..

[68] Liu W., Baker R.D., Bhatia T., Zhu L., Baker S.S. (2016). Pathogenesis of nonalcoholic steatohepatitis. Cell Mol. Life Sci..

[69] Bauerly K., Harris C., Chowanadisai W., Graham J., Havel P.J., Tchaparian E., Satre M., Karliner J.S., Rucker R.B. (2011). Altering pyrroloquinoline quinone nutritional status modulates mitochondrial, lipid, and energy metabolism in rats. PLoS ONE.

[70] Brown M.S., Goldstein J.L. (1997). The SREBP pathway: Regulation of cholesterol metabolism by proteolysis of a membrane-bound transcription factor. Cell.

[71] Uyeda K., Repa J.J. (2006). Carbohydrate response element binding protein, ChREBP, a transcription factor coupling hepatic glucose utilization and lipid synthesis. Cell Metab..

[72] Ruderman N.B., Xu X.J., Nelson L., Cacicedo J.M., Saha A.K., Lan F., Ido Y. (2010). AMPK and SIRT1: A long-standing partnership?. Am. J. Physiol. Endocrinol. Metab..

[73] Zhang J., Meruvu S., Bedi Y.S., Chau J., Arguelles A., Rucker R., Choudhury M. (2015). Pyrroloquinoline quinone increases the expression and activity of Sirt1 and -3 genes in HepG2 cells. Nutr. Res..

[74] Jonscher K.R., Chowanadisai W., Rucker R.B. (2021). Pyrroloquinoline-quinone is more than an antioxidant: A vitamin-like accessory factor important in health and disease prevention. Biomolecules.

[75] Zhang H., Li J., Cao C., Zhang B., Yang W., Shi B., Shan A. (2020). Pyrroloquinoline quinone inhibits the production of inflammatory cytokines via the SIRT1/NF-κB signal pathway in weaned piglet jejunum. Food Funct..

[76] Devasani K., Kaul R., Majumdar A. (2020). Supplementation of pyrroloquinoline quinone with atorvastatin augments mitochondrial biogenesis and attenuates low grade inflammation in obese rats. Eur J. Pharmacol..

[77] Ishak N.S.M., Ikemoto K., Kikuchi M., Ogawa M., Akutagawa K., Akagawa M. (2021). Pyrroloquinoline quinone attenuates fat accumulation in obese mice fed with a high-fat diet, *Daphnia magna* supplied with a high amount of food, and 3T3-L1 adipocytes. ACS Food Sci. Technol..

[78] Fu S., Yang L., Li P., Hofmann O., Dicker L., Hide W., Lin X., Watkins S.M., Ivanov A.R., Hotamisligil G.S. (2011). Aberrant lipid metabolism disrupts calcium homeostasis causing liver endoplasmic reticulum stress in obesity. Nature.

[79] Uhl O., Demmelmair H., Segura M.T., Florido J., Rueda R., Campoy C., Koletzko B. (2015). Effects of obesity and gestational diabetes mellitus on placental phospholipids. Diabetes Res. Clin. Pract..

[80] Galiero R., Caturano A., Vetrano E., Cesaro A., Rinaldi L., Salvatore T., Marfella R., Sardu C., Moscarella E., Gragnano F. (2021). Pathophysiological mechanisms and clinical evidence of relationship between Nonalcoholic fatty liver disease (NAFLD) and cardiovascular disease. Rev. Cardiovasc. Med..

[81] Dobrinskikh E., Al-Juboori S.I., Shabeka U., Reisz J.A., Zheng C., Marwan A.I. (2019). Heterogeneous pulmonary response after tracheal occlusion: Clues to fetal lung growth. J. Surg. Res..

[82] Folz J.S., Shalon D., Fiehn O. (2021). Metabolomics analysis of time-series human small intestine lumen samples collected in vivo. Food Funct..

[83] Cajka T., Fiehn O. (2016). Increasing lipidomic coverage by selecting optimal mobile-phase modifiers in LC–MS of blood plasma. Metabolomics.

[84] Tsugawa H., Ikeda K., Takahashi M., Satoh A., Mori Y., Uchino H., Okahashi N., Yamada Y., Tada I., Bonini P. (2020). A lipidome atlas in MS-DIAL 4. Nat. Biotechnol..

[85] DeFelice B.C., Mehta S.S., Samra S., Čajka T., Wancewicz B., Fahrmann J.F., Fiehn O. (2017). Mass Spectral Feature List Optimizer (MS-FLO): A tool to minimize false positive peak reports in untargeted liquid chromatography-mass spectroscopy (LC-MS) data processing. Anal. Chem..

[86] Reisz J.A., Zheng C., D’Alessandro A., Nemkov T. (2019). Untargeted and semi-targeted lipid analysis of biological samples using mass spectrometry-based metabolomics. Methods Mol. Biol..

[87] Nemkov T., Reisz J.A., Gehrke S., Hansen K.C., D’Alessandro A. (2019). High-throughput metabolomics: Isocratic and gradient mass spectrometry-based methods. Methods Mol. Biol..

